# Identifying left and right hemispheres using functional connectivity

**DOI:** 10.64898/2025.12.12.694044

**Published:** 2025-12-13

**Authors:** Trevor K. M. Day, Peter E. Turkeltaub, Elissa L. Newport, Andrew T. DeMarco

**Affiliations:** 1. Center for Brain Plasticity and Recovery, Georgetown University, Washington, D.C., U.S.A.; 2. MedStar National Rehabilitation Hospital, Washington, D.C., U.S.A.

**Keywords:** hemispheric organization, handedness, right-handedness, left-handedness, connectome

## Abstract

Many studies have analyzed what organizational features distinguish the left and right hemispheres of the human brain, with most differences typically being found in language areas. In this analysis, we test whether supervised learning can categorize an unseen hemisphere as right or left based on functional connectivity. Using data from the Human Connectome Project, we find success to be extremely high (accuracies > .90) in right-handed participants (Edinburgh Handedness Inventory [EHI] > 0). Accuracies are still high, but slightly lower when trained on left-handed participants (EHI ≤ 0). In a third analysis, we test whether the same can be done to identify handedness along with hemisphere chirality. This does not succeed, however, we show that individuals’ hemispheres are less distinct the more left-handed they are. Our findings can inform developmental and post-injury work on hemispheric organization.

At first glance, the two hemispheres of the human brain seem to be mirror images of each other. The gross anatomical features and gyral patterns are symmetrical to the naked eye, and the cortex is organized into about a dozen functional networks that are largely symmetric. These networks are consistently replicated across task and resting-state (RS) studies (Hermosillo et al., 2024; Ji et al., 2019; Kong et al., 2025; Power et al., 2011; Yeo et al., 2011).

However, from classic lesion work, the left hemisphere (LH) has long been known to support the language faculty (Broca, 1865; Dax, 1865; Wernicke, 1874). LH damage results in aphasia — impairment of productive and receptive language — much more frequently than damage to the right hemisphere (RH) (Dewarrat et al., 2009; Turkeltaub, 2019). The language network (LN) is left-lateralized in adults (Lipkin et al., 2022; Poeppel et al., 2012; Skeide & Friederici, 2016), and becomes more lateralized over development (Olulade et al., 2020; Szaflarski, Holland, et al., 2006; Szaflarski, Schmithorst, et al., 2006). Furthermore, there is evidence for differences in connectivity between the left and right auditory network (Mišić et al., 2018). Five major white-matter tracts connect the language areas, namely the arcuate (AF), superior and inferior longitudinal, uncinate, and inferior fronto-occipital fasciculi (Campbell & Pike, 2014; Skeide & Friederici, 2016), with the AF being the most consistently lateralized tract (Glasser & Rilling, 2008; Lebel & Beaulieu, 2009), although results vary (Thiebaut de Schotten et al., 2011).

In contrast, the RH preferentially supports visuospatial processing, including face processing (Kanwisher & Yovel, 2006; Lochy et al., 2019) and attention. The ventral attention network (VAN) is lateralized to the RH (Corbetta et al., 2008; Corbetta & Shulman, 2002) and forms an approximate “mirror” of the LH LN (Bernard et al., 2020; Day, 2024; Ji et al., 2019; Lee et al., 2012; Strike et al., 2023). Unilateral spatial neglect typically follows RH, but not LH injury (Corbetta & Shulman, 2011; Ocklenburg & Güntürkün, 2024; Stone et al., 1993). Additionally, the dorsal attention network (DAN) is also right-lateralized (Wang et al., 2014) and the RH halves of these RSNs are more variable compared to the LH (Perez et al., 2023; Seitzman et al., 2019), suggesting differences in broad connectivity patterns between the two hemispheres. Finally, the frontoparietal network (FPN) connects preferentially to different networks in each hemisphere (Wang et al., 2014).

## Handedness

In the majority of adults, the LH is responsible for fine motor control of the hands, such as writing or throwing a ball. Globally 90% of the population is right-handed (Papadatou-Pastou et al., 2020), and the majority of neuroscience research has been focused on them because left-handed individuals (or “sinistrals”) are known to be more variably organized cortically than right-handers (“dextrals”), see Willems et al. (2014) for a discussion. However, the relationship between handedness and lateralization is not clear. Sinistrals are not the opposite of dextrals in cortical organization. While most sinistrals are left-lateralized for language, there is a somewhat higher rate of bilateral and right-dominant distributions of the LN in this population (Knecht et al., 2000; Szaflarski et al., 2002). There is conflicting evidence on structural differences in the brain between sinistrals and dextrals, but the largest study to date (*n* dextral = *28,802; n* sinistral = *3,062)* detected small differences in surface area and thickness between the groups, especially in language and executive function regions (Sha et al., 2021), and there are differences in structural network composition between sinistrals and dextrals (Caeyenberghs & Leemans, 2014).

## Hemispheric flexibility

At birth, the brain displays incredible plasticity. In language processing, children who have a perinatal stroke to the LH LN show a remarkable capacity to reorganize language processing to the RH homologues and generally show normal language skills (Martin et al., 2023; Newport et al., 2022; Seydell-Greenwald et al., 2025). However, the same is not true for adults, and while some changes occur in the contralesional hemisphere following a unilateral stroke, the degree to which the uninjured hemisphere reorganizes is an active area of work (Billot & Kiran, 2024; Makin & Krakauer, 2023; Turkeltaub et al., 2025; Turkeltaub & Martin, 2024; Wilson & Schneck, 2021). The features of hemispheric organization that enable this plasticity in early, but not later life, remain under investigation.

## Classification

Lateralization is an important feature of cortical organization, against the broader backdrop of a mostly symmetric cortex. Naturally, most prior work has focused on testing differences between individuals’ hemispheres. Here, we ask whether the hemispheres are distinct enough to permit the classification of an unknown hemisphere as left or right. Developing a classifier for hemispheres might reveal which within-hemisphere network features support hemispheric specialization. Furthermore, such a classifier would permit future work to ask how a developing hemisphere becomes more adult-like in its organization over age; or how an hemisphere contralateral to an injured one is organized after a stroke, e.g. is it more like a typical right or left hemisphere?

Resting-state organization has previously been used to identify individuals out of a group (Finn et al., 2015; Hannum et al., 2023; Kardan et al., 2022; Miranda-Dominguez et al., 2014), i.e., one-out-of-many classification, typically called “fingerprinting.” Cortical organization can also be used to identify task state within an individual, i.e., many-to-one (Hannum et al., 2023) and predict predict continuous variables, e.g., age in young children (Kardan et al., 2022). Here, we use supervised learning to test whether the chirality of a hemisphere is identifiable. Because the broader questions about hemispheric specialization involve participants who may struggle with certain tasks (e.g., young children, stroke survivors), we use resting-state connectivity, which can be applied in a wide range of populations.

## Current Analysis

We use three types of supervised learning — linear discriminant analysis (LDA), support vector classification (SVC), and neural nets (NN) — to attempt to classify hemisphere chirality from connectivity data. Our primary focus is resting-state fMRI (rsfMRI) data, which is generalizable and collected in many modern studies. Because auditory and language processing is strongly lateralized, we also examine connectomic features calculated from scans collected while participants performed a language task.

This paper is structured in three major analyses. The first attempts to classify partial connectomes as belonging to a right or left hemisphere in a large group of dextral individuals. The second conducts the same analyses in a group of sinistral individuals. Finally, the third then attempts to classify partial and complete connectomes as belonging to a sinistral or dextral individual, and the success and weaknesses of these approaches are compared.

We hypothesized that the models would be able to identify hemisphere chirality, at least in dextrals, with no a priori hypotheses about which kind of model might perform best. We anticipated that hemispheric classification would be more accurate than handedness classification. Finally, we anticipated that connections between auditory and language areas would drive the differences between the hemispheres.

## Analysis 1: Hemispheric classification in dextrals

### Methods

Our goal is to predict hemispheric chirality from within-hemisphere connectomes, “hemiconnectomes.” Based on the highest-performing methods on a similar task, from Hannum et al. (2023), we used three modeling approaches to classify hemiconnectomes: linear discriminant analysis (LDA), support vector classification (SVC)^1^, and a neural net (NN)^2^, all implemented in the Python 3 package scikit-learn, ver. 1.6.1 (Pedregosa et al., 2011).

#### Participants

We begin with the larger group of dextral participants in HCP, which is consistent with earlier work in neuroscience, and avoids introducing the more heterogenous and much smaller sinistral group. To select dextral individuals, we used an Edinburgh Handedness Inventory score (Oldfield, 1971) greater than 0, *n = 875*.

#### Image acquisition

Minimally preprocessed data was accessed from the HCP repository, ConnectomeDB (db.connectome.org). The acquisition parameters and minimal preprocessing is described in Van Essen et al. (2013) and Glasser et al. (2013). We retrieved the resting state (RS) and language task (LgT) data, both of which were acquired with a TR of 0.7 s, and 2 mm isotropic voxels. For each participant, two 15 min RS scans (total: 30 min) were acquired as well as two 3:47 LgT scans (total: 7:34)

Because language processing is known to be strongly lateralized, we also analyze the BOLD data collected during the LgT. The HCP LgT interleaves auditory story blocks (5 – 9 sentences; average duration: 29.8 (14.5) sec) and math blocks (duration varied based on difficulty). After each block, the participant responded to an 2AFC question about the topic of the story or the answer to the math problem (Barch et al., 2013; Binder et al., 2011).

#### Atlas selection

We use the Glasser atlas (Glasser et al., 2016), which has 360 parcels, 180 in each hemisphere, resulting in 64,440 unique parcel-to-parcel connections. These connections comprise the full connectome. We divide the full connectome into two “hemiconnectomes” (HCs) consisting of only within-hemisphere connections, each *n = 16,110*. All 180 parcels appear in both hemispheres.

To aid in interpretation, we use RSNs created from the Glasser atlas and HCP-YA data. Each parcel is assigned to one RSN based on the Cole-Anticevic RSNs (Cole & Ito, 2018; Ji et al., 2019). The Cole-Anticevic networks are not perfectly symmetric, and when homologous parcels were assigned to different networks, the LH assignment was selected to maximize the size of the LN, *n = 14*. Five parcels were assigned to the LN in the LH, but different networks^3^ in the RH, see Supplementary Material *YY* for more detail. No parcels were assigned to the LN in the RH, but not the LH.

Of the 12 Cole-Anticevic networks, we merge their two visual networks. The small posterior and ventral multimodal (PMN, VMN) and orbito-affective networks (OAN) are ignored (all with three or fewer parcels). The auditory network (AudN) is also small, but kept, due to its relationship with the lateralized LN, see Table M1.

#### Preprocessing

Data were further preprocessed using the eXtensible Connectivity Pipeline-DCAN, (XCP-D) ver. 0.10.6 (Ciric et al., 2018; Mehta et al., 2024; Salo et al., 2025; Satterthwaite et al., 2013). The automatically generated description of the processing is shared below. This description was edited for clarity (additionally, see Salo, 2025).

First, the motion parameters were calculated. The six translation and rotation head motion traces were low-pass filtered below 6.0 breaths-per-minute using a second-order Butterworth filter, based on Gratton et al. (2020). The Volterra expansion of these filtered motion parameters was then calculated. Framewise displacement (FD) was calculated from the filtered motion parameters (Power et al., 2014), with a head radius of 50 mm. Volumes with filtered FD values greater than 0.2 mm were flagged for the sake of later censoring (Power et al., 2014). In total, 27 nuisance regressors were used: the (#1 – 6) six motion parameters with their (#7 – 12) temporal derivatives, and (#13 – 24) the quadratic expansion of those 12 values; and (#25) mean global signal, (#26) mean white matter signal, and (#27) mean cerebrospinal fluid signal (Ciric et al., 2017; Satterthwaite et al., 2013). All motion parameters were filtered using the same parameters as described above and the Volterra expansion was calculated. The BOLD data were converted to NIfTI format, despiked with *AFNI*’s *3dDespike* (using the default settings, no mask, and the “NEW” option), and converted back to CIFTI format.

Nuisance regressors were calculated from the BOLD data, replicating *Nilearn*’s approach (Lindquist et al., 2019; Nilearn developers, 2024b; Salo, 2025), with the following exceptions. (1) Any volumes censored earlier in the workflow were first cubic-spline interpolated. (2) Outlier volumes at the beginning or end of the timeseries were replaced with the closest low-motion volume’s values using *Python*, to avoid extreme interpolations induced by cubic-spline interpolation. The resulting timeseries were band-pass filtered using a second-order Butterworth filter implemented in *Nilearn* (Nilearn developers, 2024a), in order to retain signals between 0.01 – 0.08 Hz. The same filter was applied to the confounds.

After these steps, the band-pass-filtered timeseries were denoised using linear regression in Numpy (Harris et al., 2020). For this calculation, only the low-motion volumes were used to calculate parameter estimates. The interpolated volumes were denoised using the parameter estimates calculated from the low-motion volumes. The interpolated timeseries were then censored using the FD values calculated initially.

Next, the denoised BOLD was then smoothed using *Connectome Workbench* (Marcus et al., 2011) with a Gaussian kernel (FWHM = 6.0 mm). Average within-region timecourses were extracted from the cleaned and filtered BOLD data using *Connectome Workbench*, using regions defined by the Glasser atlas. Pairwise functional connectivity (Pearson’s *r*) between all regions was computed using *Connectome Workbench*. In cases of partial coverage, uncovered vertices (values of all zeros or NaNs) were either ignored (when the parcel had > 50% coverage) or were set to zero (when the parcel had < 50% coverage).

Many internal operations of *XCP-D* use *AFNI* (Cox, 1996; Cox & Hyde, 1997)*, Connectome Workbench* (Marcus et al., 2011), *ANTS* (Avants et al., 2009)*, TemplateFlow* version 24.2.2 (Ciric et al., 2022)*, matplotlib* version 3.10.0 (Hunter, 2007), *Nibabel* version 5.3.2 (Brett et al., 2022)*, Nilearn* version 0.11.1 (Abraham et al., 2014), *numpy* version 2.2.1 (Harris et al., 2020), *pybids* version 0.18.1 (Yarkoni et al., 2019) and *scipy* version 1.15.1 (Harris et al., 2020). For more details, see the *XCP-D* website (https://xcp-d.readthedocs.io).

Before model training, Pearson’s *r* values were Fisher-Z transformed. We also calculated hemiconnectomes from the LgT data (LgHC), processing it identically, ignoring the task structure. We anticipated that the increased connectivity in language areas due to a task that creates lateralized activation provides increased information for the models to identify hemisphere chirality by increasing the magnitude of lateralized connections during a task by increasing blood flow to those areas, which is measured during rsFMRI.

#### Model assessment

For reliability, the dataset was separated into five test groups (“folds”) based on handedness status. Each analysis was repeated for each fold, i.e. test fold A was tested against models trained on folds B-E, such that each individual is trained on four times, but only tested once.

We need a single value to assess model performance, both between models and relative to random chance. Rather than raw classification accuracy, we use Matthew’s correlation coefficient (MCC) to characterize model performance. MCC, like accuracy, sensitivity, or specificity, operates on confusion matrices, but falls on [−1, 1] and is scaled such that random assignment in one class or more is always MCC = *0* (Chicco & Jurman, 2023; Rainio et al., 2024; Yilmaz & Demirhan, 2023). We use MCC rather than accuracy, because the latter can be misleading in analyses with imbalanced classes, as in Analysis 3. For example, if the model assigns all participants to be dextral, even if the 10% of the sample was sinistral, accuracy would be 90%, which is misleading, since the model is wrong about half of the data types.

There is no analytic standard for calculating *p* values for MCC. MCC is sensitive to class imbalances, e.g. in the case that an entire group is misclassified, the MCC will be 0. However, the relationship is not linear, and simulations suggest that for the present analysis, MCCs of 0.4 and 0.6 are approximately equivalent to 80% and 90% accuracy in each class, see Supplementary Material ZZ. For all analyses, we adopt these critical values of MCC = {0.4, 0.6} as benchmarkers for “good” and “excellent” performance, respectively.

For each set of model results, we bootstrap a 95% confidence interval for that model’s MCC by randomly sampling the ground truth and predicted labels with replacement to generate a distribution of MCC, *n = 1,000* repetitions. The mean of this distribution is taken as the MCC value, and the 2.5th and 97.5th percentile of this distribution is used as the 95% confidence interval for the MCC value.

#### Identification of important connections by network

In order to identify what kind of networks are important, we follow an “enrichment” approach, similar to the one described by Eggebrecht and colleagues (2017). There are 16,110 features in each model, and each feature in the model is a functional connection between two parcels. LDAs provide the most interpretable parameters for the classification problem, as each feature is given a scaling that represents its importance for predicting the outcome. As such, each feature can be a within-network connection, or a between-network connection.

First, we identify significant features using permutation testing. We randomly shuffle the labels on the input data and re-run the models on this data (“null models”). We repeat this process 10,000 times and extract the feature scalings for the null models, calculate the empirical significance level for the true feature scaling against these repetitions, using the get_p_value() function from the R package infer (ver. 1.0.8; Couch et al., 2021), i.e., a two-sided rank proportion test.

Following this test, connections are marked as significant if the uncorrected *p*-value is less than 0.01. Then, we permute a null distribution of connections across the brain by randomly shuffling those significant values across the connections. Connectome matrices are symmetric, thus our permutation procedure also maintains the symmetry of the matrix. We then summarize the number of significant connections by the network pair (or single network) they belong to, and again use get_p_value() to establish if there are more significant connections in that network pair than expected by chance.

However, it is important to note that feature scalings (i.e., connection weights) do not straightforwardly represent “feature importance” or whether the connection is stronger in the LH or RH. In short, connections that are important to the model, but where the difference between the hemispheres is smaller in magnitude, the scaling will be greater in order to load onto the model. This is addressed in the bootstrapping procedure. Furthermore, the sign of the scaling is arbitrary, but similar connections cluster together within each sign group.

Each pair of networks (or set of within-network connections receives a single *p* value that is later corrected for multiple comparisons. We use false-discovery rate (FDR) correction for 36 multiple comparisons. Simulated *p*-values of 0.0 are replaced with values of .0003 for correction, based on information from the infer manual^4^, which states that the minimum simulated *p*-value scales with *3 / repetitions*, which was 10,000.

#### Sensitivity analyses

Although the Glasser atlas contains parcels that are exactly matched across the hemispheres, they are not identical in extent. The authors state that “a few areas show hemispheric asymmetries in their functional ‘signature’ and/or in their spatial relationships with neighbouring areas” (p. 179), and elsewhere state that the main asymmetries are in the language areas. To test whether the model outcomes are dependent on differences in region borders that differ between the hemispheres, we mirror the parcellation and use that as input to the training models. We term this model “*HC-R*” (Reversed).

Along with the HC-R test, we perform two additional sensitivity analyses. In the network analysis step, test permutations that re-include the excluded networks (Supplementary Materials XX.1); and separately, exclude the ROIs that are part of different networks in each hemisphere (Supplementary Materials XX.2). The former analysis expands the permutation space, and the latter tests that the networks identified as contributing to the differences between the hemispheres are not different only because they included some ROIs, but not others, in the RH.

#### Data availability

All data are publicly available through the Human Connectome Project or Zenodo (for the parcel-to-network assignments).

### Results

#### Model performance

**Figure R1:**
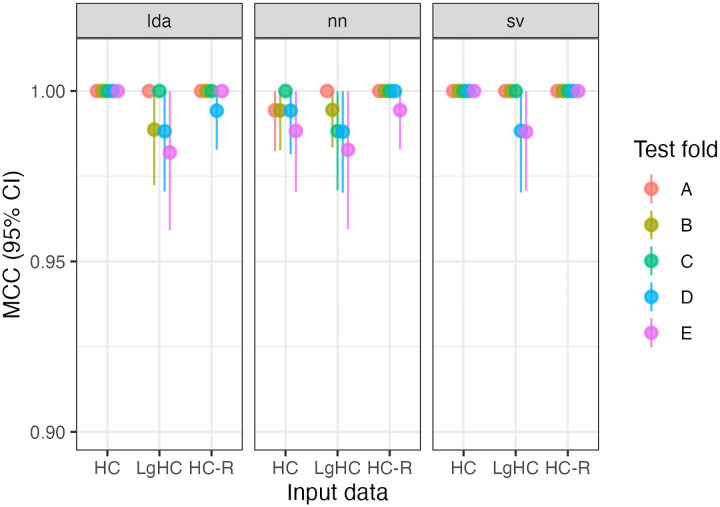
Model performance for five folds (A-E) across three model types (LDA, NN, SVC) and two input data sets (resting-state hemiconnectome: HC; language task hemiconnectome: LgHC; and reversed parcellation: HC-R), totalling 45 models.

All five folds trained on RS data perfectly classify (i.e, MCC = 1) hemisphere chirality for LDA and SVC. For the NN, MCC_NN_ = [.994, 1]. All three kinds of models also perform extremely well when trained on LgT data: MCC_LDA_ = [.983, 1], MCC_NN_ = [.982, 1], MCC_SVC_ = [.988, 1] with single-digit errors out of 875 predictions. Reversing the parcellation did not affect model performance, with SVC perfectly classifying the hemispheres, and MCC_LDA_ = [.994, 1], MCC_NN_ = [.995, 1]. See Figure R1. All of these MCC values are equivalent to accuracies greater than 0.99.

Based on these results, we limited further analyses to the resting-state state data, parcellated according to the canonical (i.e., unreversed) Glasser atlas (label: HC). Furthermore, we focused on the LDA models, which allow for the most straightforward interpretation. We investigated the linear discriminant (LD1) values for each of the hemispheres for a new model trained on the entire sample (n = 935). Because we are no longer testing model reliability, and instead examining model features, we train a maximal model on all participants, since model features cannot easily be compared between the five-fold models.

**Figure R2:**
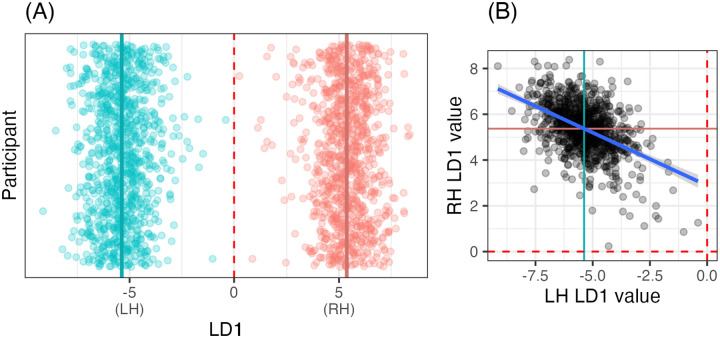
**(A)** Linear discriminant (LD1) values by hemisphere (red: RH; blue: LH) and participant (*y*-axis). Each hemisphere clusters around an LD1 value of approximately −5 or +5. (B) Correlation between LD1 values for each hemisphere. Means for both hemispheres are given in both subfigures as a dark colored line; and 0 is marked in both subfigures as a dashed red line.

In this maximal model, none of the hemispheres were misclassified, see Figure R2(A), where each hemisphere clusters around an LD1 value of −5 (LH) or 5 (RH). Per-hemisphere LD1 values are correlated (*r = −.47, p < .001)*, in such a way that if a participant had one hemisphere closer to LD1 = 0, the contralateral hemisphere was likely to be closer to 0 as well, see Figure R2(B).

#### Enrichment results

**Figure R3:**
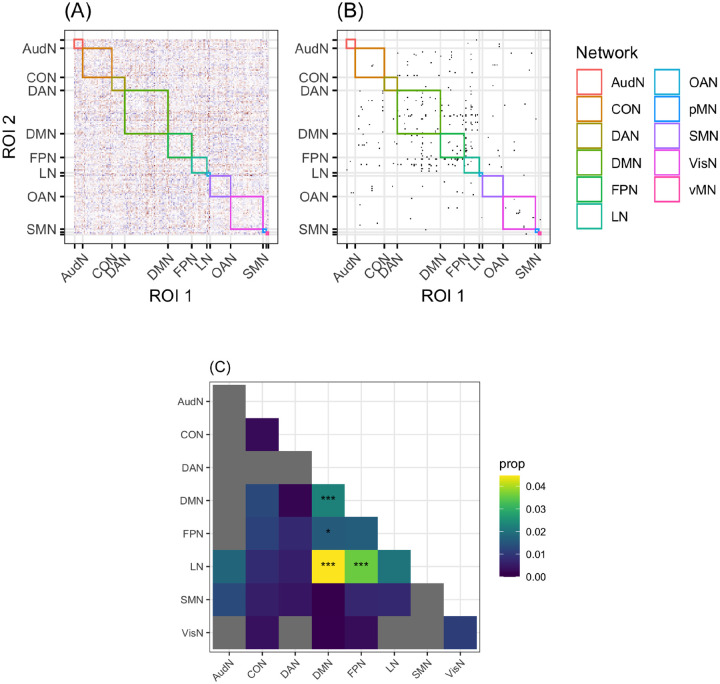
An illustration of the enrichment analysis. Panel A shows the scaling values for each connection for all networks, grouped by network label. Panel B shows which of these values are higher than expected by chance (using bootstrapping for significance), and panel C shows which network pairs (or single networks) have more significant connections than expected by chance (using bootstrapping for significance), marked by asterisks: *: uncorrected *p* < .05, ***: uncorrected *p* < .001. Networks with no significant connections (after step B) are in grey, and networks are colored by the proportion of connections within that group that are significant, even if there aren’t more than expected by chance.

The purpose of the enrichment analysis was to identify what kind of connections (i.e., between which networks) permit the classification of a hemisphere as left or right. Figure R3 illustrates the enrichment process.

Four network groups (within-DMN, DMN-FPN, DMN-LN, and FPN-LN) had more connections than expected by chance, *p < .05*. The DMN-FPN group is no longer significant after correction, *p*_*cor*_ = *.12*. The *p*_*cor*_ values for the remaining three relations were all .0036. The features that are most important to the model are primarily connections that are stronger in the LH relative to the RH (*n = 83*), but not exclusively so (RH > LH *n = 49*). No significant connections are found in the AudN, and connections within the LN do not explain the differences between the hemispheres. The results survive two sensitivity analyses (1) evaluating the excluded networks (SM XX.1) and (2) excluding the ROIs that are given different network labels in each hemisphere (SM XX.2)

**Figure R4:**
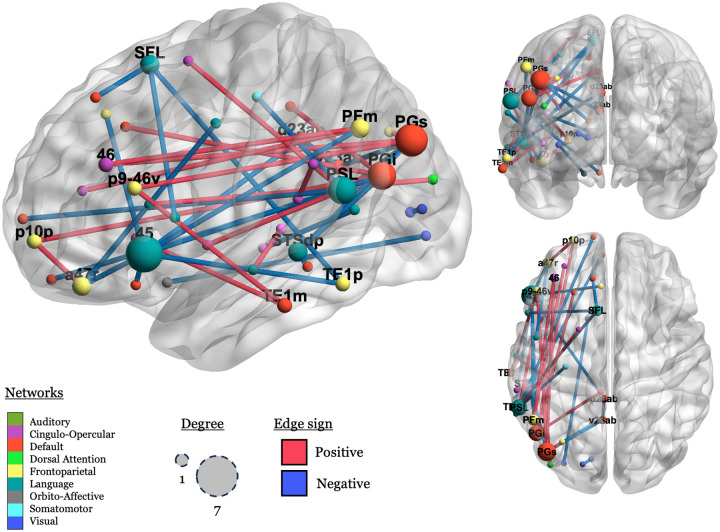
Visualization of significant connections and their associated nodes (ROIs). Nodes (spheres) are scaled by the number of significant connections they are involved in (1 – 7), and nodes with degree > 1 are labeled. Edges are colored by sign (blue: negative; red: positive). Nodes are colored by network, using the colors used in Ji et al. (2019), with some modifications. Node names are from Glasser et al. (2016).

Finally, we visualize the significant connections in order to illustrate which ROIs had any important connections to them, defined as a connection with a permuted *p* value < .001. There were 39 connections that met that threshold, connecting 44 regions in nine networks, see Figure R4.

In Figure R4, we can see that negative (blue; LH > RH) connections predominate in connections to the LN, and that positive (red; RH > LH) connections predominate in more ventral connections between a more diverse set of networks, including CON, FPN, and the DMN.

Inspecting the spatial distribution of the important connections, language regions like BA 45 (label: “45”), the peri-sylvian language area (“PSL”), and the superior frontal language area (“SFL”), emerge as important nodes, but the important connections are mostly between these areas and non-language networks, as previously discussed.

### Analysis Summary

A complete discussion of the three major analyses comes at the end of the paper. What follows is a brief summary of the analysis including only dextrals. Our results show that, for dextrals, within-hemisphere connectivity is strikingly distinct, with some models never erring on classifying an unseen hemisphere’s chirality. These results are robust across methodologies and input data and replicate across folds. Overall, the language task data results in slightly poorer predictions than the resting-state data but still nearly at ceiling, with remarkably few errors, which suggests that classification can be performed with less than 8 minutes of data, rather than the 30 minutes used by the resting-state analysis.

## Analysis 2: Hemisphere classification in sinistrals

### Methods

One potential challenge of analyzing sinistrals is that their cortical organization is known to be less strongly lateralized (Knecht et al., 2000; Szaflarski et al., 2002; Van der Haegen & Brysbaert, 2018). Analysis 2 sought to test whether the additional inherent variability in sinistrals would hurt model performance, relative to the dextrals in Analysis 1. To address these questions, the connectome-preparation and model evaluation steps from Analysis 1 are repeated, except with the HCP individuals with an EHI ≤ 0, *n = 92*. This groups’ hemiconnectomes are termed “HC (L).”

The sinistral group is much smaller than the dextral group, which raises questions about whether potential penalties in performance are attributable to the smaller sample size, rather than greater heterogeneity in the sinistrals. To control for sample size, we additionally create comparably sized samples from the dextral group to test the effect of input data size. For each fold, we sample 74 ($\frac{4}{5}\text{ths}$ of 92) dextral training participants from the training groups (e.g., training folds A-D) and test them on the full held-out training fold (e.g., test fold E, *n* about 200). Testing on the entire held-out training fold prioritizes comparing MCC values between dextral groups, matching the sinistral test fold size (about 20) would magnify classification errors in the numerator when the denominator is $\frac{1}{10}\text{th}$ of the size.

These control groups separate the effects of (1) training on a smaller sample; from (2) training on less-dextral participants. We evaluate the effect of sample size by using a paired *t*-test to evaluate performance, as measured by MCC, for the HC74 samples against their paired full HC models trained on dextrals only, and the corresponding models trained on sinistrals only. We predicted that differences in chirality classification success would be primarily attributable to handedness status, rather than training group sizes.

### Results

#### Model performance

**Figure R5:**
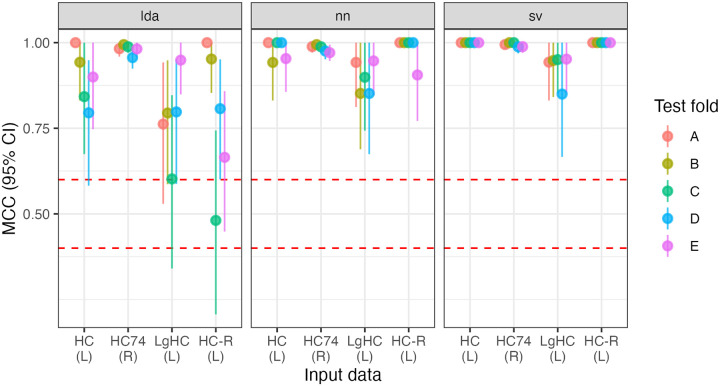
Model performance for sinistral participants (L) and the dextral comparison group ^®^, across five folds (A-E) across three model types (LDA, NN, SVC) and two input data sets (resting-state hemiconnectome: HC; language task hemiconnectome: LgHC; and reversed parcellation: HC-R), totalling 45 models. Small groups of dextral training groups are included for comparison (“HC74 (R)”). The MCC “good” and “excellent” thresholds are included at MCC = 0.4 and 0.6.

Generally speaking, training on only the sinistrals performs above the “excellent” threshold, but not as high as the dextral-only group. That is, there is still very high identification of LH vs. RH based on connectivity patterns, see Figure R5.

First, variance in MCC values are higher in this sample compared to the dextrals, so we test, among the sinistral training data, if the classification method (LDA, SVC, NN) and input data (HC, LgHC, HC-R) affect MCC. Using a linear mixed-effects (R package lmerTest, ver. 3.1–3) model (with test fold as the random effect), we find that there is no effect of model (*p*s > .07), and that the LgHC and HC-R data under-perform the base data (*β = −.11* for both, uncorrected *p = .047*, for both). None of the interaction terms were significant.

Second, we test whether model performance in sinistral and dextral groups differs between models or input data type. In terms of fixed effects, although the folds are labeled the same in both the dextral and sinistral analyses, they share no participants. Thus, the random effect separates the folds by handedness status. Because there was significant variability in Figure R5, we test a saturated model of all interactions between handedness (sinistral/dextral), method (LDA, SVC, NN), and input (HC/LgHC). There was a main effect of handedness, *β*_*R>L*_ = *.10, p = .001*. Additionally, both the NN and SVC models out-performed LDA, *β*_*NN>LDA*_ = *.08, p = .006, β*_*SVC>LDA*_ = *.10, p < .001*. There was a main effect of input, with LgHC leading to lower MCC, *β*_*LgHC>HC*_ = *−.11, p < .001*. There was a significant interaction between dextrality and LgHC, *β = .11, p = .012,* and between dextrality and both NN>LDA (*β = .09, p = .035*) and NN>SVC (*β = .10, p = .015*).

Finally, we separately test whether the MCC values are smaller in the subsetted dextral group (range: [.956, 1.0]) compared to the complete dextral group (range: [.988, 1.0]). We run a paired *t*-test between all 15 MCC values (five folds ✕ three models) for the base data. The reduced sample results in lower MCC values, mean difference: −.012, *t(14) = −4.2, p = .009, d = 1.1*.

The HC-R training data also appeared to be affected by the model used. Inspecting Figure 5, the LDA appeared to do much more poorly compared to SVC and NN. Repeating the mixed-effects model, using only the training model as a covariate, both SVC and NN outperformed LDA, *β*_*SVC>LDA*_ = *.20, p = .032*, but the NN did not, *β*_*NN>LDA*_ = *.22, p = .02*.

In summary, training models on the sinistrals results in lower average performance, but this decrease is mostly isolated to the LDA model and in the LgHC data. Some small proportion (approximately 10%) of the lower performance could be attributed to the decrease in training sample size. We can also note that performance using the HC-R input data was also lower for the LDA, but not the other tasks, although we did not include the sensitivity test in the main models.

#### Feature exploration

A repetition of the enrichment analysis on the sinistral data results in a very similar pattern as the one found in the dextrals, i.e., the most important connections that define the differences between the hemispheres are found in connections between LN-DMN and connections between LN-FPN. However, within-FPN and within-LN connections are important for sinistrals (where they were not for dextrals), and DMN-FPN connections are not important for sinistrals (where they were for dextrals), see Supplementary Figure XX.3.

There were 64 connections identified as important (*p < .001*; dextrals: 39) that connected 66 (dextrals: 44) unique areas in nine networks (dextrals: 9). All 44 areas that were important in the dextral-only analysis were found in the sinistral-only analysis. Those 22 additional areas were found in the DMN (n=6), CON (5), FPN (4), SMN (4), and VisN (3). A similar ball-and-stick model is shown below.

### Analysis Summary

This analysis replicates and extends the findings of Analysis 1 to individuals known to be more variable in their cortical organization than dextrals. The models trained on sinistrals are somewhat lower and more variable in performance, but nearly all perform with excellent (MCC > .6) performance, which was affected slightly by the decrease in model training data.

Notably, the LDA models under-perform relative to SVC and NN. We still use it to examine hemisphere “distance” scores, which requires more connections (*n = 66*) relative to the dextral-trained model (*n = 44*), although a reliance on *more* features in the face of *less* data is not surprising, and all 44 dextral features appear in the sinistral set of 66. As in Analysis 1, the language task under-performs relative to the rs-fMRI data. The primary drawback of those data (reduced minutes of acquisition) are also present, but it is also possible that reduced left-lateralization among sinistrals during a language task is reflected in impaired hemisphere classification performance when using those data.

## Analysis 3: Handedness classification

### Methods

Because of the differences in cortical organization and structural features between dextrals and sinistrals, we sought to ascertain if connectomes could be used to identify handedness as an outcome, in addition to hemisphere chirality.

The same input and preprocessing steps were taken as described in Analyses 2 and 3. However, for determining handedness, it is now possible to also test the performance of two additional sets of input data: (1) the full connectome (FC), as well as (2) the interhemispheric connections excluded from the hemiconnectomes (the “transconnectome,” or TC). These new sets of input data ask whether (1) there is information that distinguishes sinistrals/dextrals in the whole-brain pattern of connectivity; and (2) whether there is information in the cross-callosal connections that distinguish sinistrals/dextrals.

#### Class imbalance

Predicting handedness presents an imbalanced-classes problem (Lemaître et al., 2017; Yang & Wu, 2006), because only 10% of the sample is sinistral. Generally speaking, the risk is that the model overfits to the majority class (i.e., dextrals), which could reach accuracies of 90%. MCC ameliorates this risk, by assigning an MCC of 0 if any class, no matter the size, is completely incorrect (see Supplementary Material ZZ).

One method for addressing imbalance data is oversampling, i.e. duplicating rows until equal class sizes are achieved. We created a dataset (Hemiconnectome Oversampled; HCO) that oversampled sinistral participants using the RandomOverSample() function (sampling strategy: “auto”) from the Python package Imbalanced-learn (ver. 0.13.0; Lemaître et al., 2017). Because the oversampling did not improve model performance in HC, we did not oversample the other input data.

#### Outcomes

For the hemiconnectomes (HC, LgHC, and HCO), we predict a four-way outcome: LH vs. RH crossed with sinistral vs. dextral, example prediction class label: *rightyLH*. We then extract handedness (*righty*) and chirality prediction (*LH*) from the prediction label and examine performance for them separately. For FC and TC, we predict only handedness because these data include both hemispheres, and neither hemisphere, respectively.

#### Feature exploration

We wanted to be able to consider how similar one hemisphere is to a canonical left or right hemisphere, and by extension, how distinct an individual’s hemispheres are. To do this, we needed to operationalize “distance,” where larger values represent hemispheres farther apart from each other in a “hemisphere space.” To create this scale we use linear discriminant (LD) scores from the LDA model trained on all participants.

First, we create *Z(LD1)* values within-hemispheres to evaluate the distribution of LD1 values between the hemispheres. We use a one-way ANOVA to test whether *Z(LD1)* values differ for sinistrals vs. dextrals, i.e. do sinistrals have hemispheres more (*Z(LD1) > 0*) or less distinct (*Z(LD1) < 0*) on average?

Secondly, it is clear LD1 separates the hemispheres, with extreme values representing each hemisphere more strongly, and that values closer to zero represent organization more like the opposite hemisphere. Therefore, we calculate summary separation-between hemisphere values as:

1. $$\text{LD}1_{\text{dist}}=\left|\text{LD}1_{\text{LH}}\right|+\left|\text{LD}1_{\text{RH}}\right|$$

Using Equation 1, larger values represent hemispheres farther apart from each other on the LD1 axis, i.e. “hemisphere space.”

We then evaluate LD1_dist_ against continuous handedness scores, i.e. EHI scores. Because handedness is not normally distributed (it has a strong left skew, sometimes called a “J-shape”), we anticipated that a basic linear regression would not adequately capture the relation. We considered two advanced functional forms. (1) If the difference between the hemispheres is dependent on *strong hand dominance* (high absolute EHI scores), then we expect a U-shaped curve, so we test quadratic models. (2) If the difference between the hemispheres is *dependent on handedness groups*, i.e., dextral and non-dextral natural classes, then we expect different slopes in the sinistral and dextral groups, so we test segmented linear regression (Muggeo, 2016) models.

### Results

#### Handedness identification

We start by reporting the model success rates. None of the models (*n = 75*) succeed in identifying handedness, with average MCC falling between [−.073, .364], with none of the handedness-prediction models passing the “good” threshold of MCC > .4, see Figure R6.

In a repeated-measures ANOVA, there are effects of the test fold (i.e., fold repetitions A, B, C …), *F(4, 64) = 19.1, p < .001* and input data (HC, FC …), *F(4, 64) = 13.3, p < .001*, but not of modeling approach, *F(2, 64) = 2.1, p = .14*. However, all of these models can be considered to fail the handedness identification task.

#### Feature exploration

**Figure R7:**
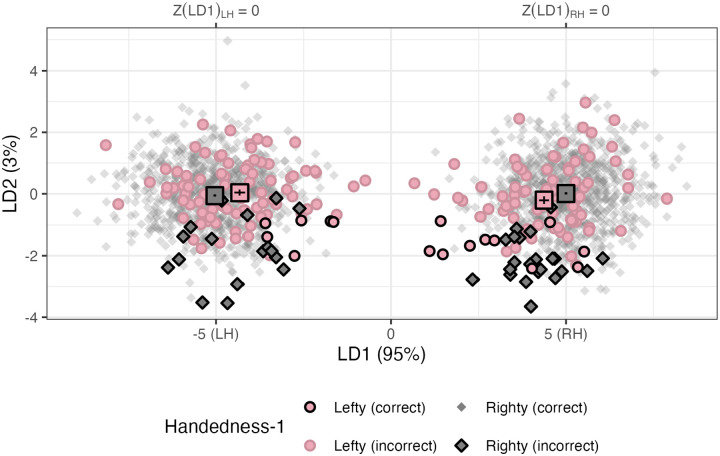
First and second linear discriminants for the four-outcome model predicting hemisphere and handedness status. The Z(LD1) transformation is marked on the upper *y*-axis for clarity. Dots are filled by actual handedness (grey/black: dextral; pink: sinistral). Hemispheres predicted to be sinsitral, regardless of identity, are outlined in black, i.e. “Lefty (correct)” and “Righty (incorrect).” The average LD1 and LD2 values for each handedness group are marked with rectangles.

As with the hemisphere models, we can explore the LDA feature values. The number of LDs is equivalent to the number of predicted classes, minus 1. This model has four classes (LH/RH and sinistral/dextral), so this model has three LDs, compared to one LD (based on two classes: LH/RH) previously. Now, we plot the first (LD1) and second linear discriminants (LD2) against each other. These LDs explained 95% and 3% of the variance, respectively. Sinistrals and dextrals cannot be separated on these axes, see Figure R7, However, we can note that sinistrals have hemispheres that are, on average, closer in LD1 (or “hemisphere space”), i.e., the horizontal distance between the rectangular centroid markers in Figure R7 is significant in both hemispheres, *β*_*LH*_ = *0.709, t(105.0) = 4.6, p < .001; β*_*RH*_ = *−0.628, t(104.9) = −4.2, p < .001*.

Like the previous model, LD1_LH_ and LD1_RH_ scores are correlated, at *r = −.07, p = .044* for the dextrals, but for sinistrals, the relationship is stronger, *r = .471, p < .001*. In other words, a sinistral with one hemisphere more like the contralateral organization (i.e., an LD1 value nearer to zero) is more likely to have the contralateral LD1 value be nearer to LD1 = 0.

**Figure R8:**
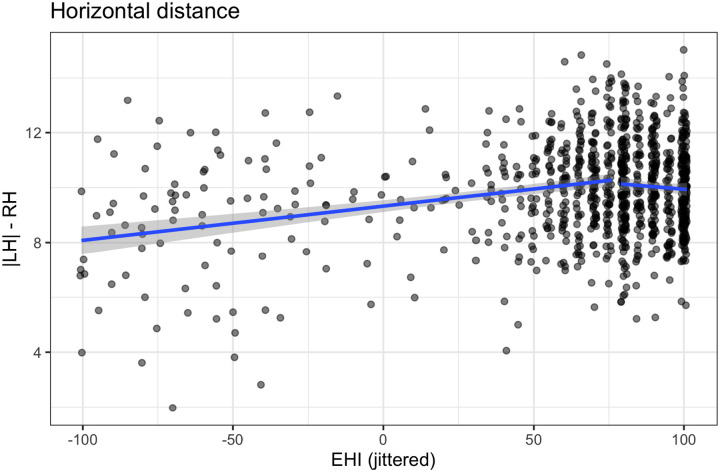
LD1_dist_ scores for all individuals, plotted against handedness score (EHI). The best-fitting, one-breakpoint model is overlaid in blue.

We evaluate *LD1*_*dist*_ scores against EHI. Using an ANOVA for linear model fits, a one-breakpoint segmented linear regression (Muggeo, 2003, 2008) improves over the simple linear model, *F(3) = 20.0, p < .001*. The breakpoint falls at *EHI = 75* (SE: *7.5*). The *R*^*2*^ for this model is 0.06, see Figure R8. The AIC for the simple linear model was 3,775 and for the one-breakpoint segmented linear regression, 3,723, an improvement of 53. A two-breakpoint does not improve over the one-breakpoint model, *F(2) = .003, p = .99*).

The quadratic model also improves over the linear model, *F(2) = 27.7, p < .001*, and the AIC for the quadratic model is 3,725, nearly identical to the AIC for the segmented linear regression model. This equation results in a downward-opening parabola with a peak at (90.9, 10.1), i.e., individuals with an EHI of about 91 have the most distinct hemispheres on average. See Supplementary Material AA.

### Analysis Summary

Overall, our functional connectivity analyses fail to distinguish sinistrals from dextrals. This failure cannot be attributed solely to the smaller training group, as hemisphere classification on dextrals succeeds with groups of similar size. Although the literature has found structural differences between sinistrals and dextrals (Caeyenberghs & Leemans, 2014; Sha et al., 2021), our findings suggest that these differences are not reflected in functional connectivity.

Our methods seek to separate within-hemisphere connectivity and connectivity related to handedness by modeling a four-way distinction. However, it is important to note here that the discrete handedness groups are created out of a continuous measurement, the EHI, which does not show discontinuities around EHI = 0. We chose an EHI cutoff of 0, but other cutoffs between dextrals and “non-dextrals” have been proposed.

That said, the models were still able to identify hemisphere chirality with near-perfect accuracy, even in the sinistral participants (and even when the handedness was identified incorrectly), suggesting that the differences between the hemispheres are larger and more stable than those between dextrals and sinistrals.

## Discussion

Our goals were to use supervised learning models to test whether it was possible to identify a hemisphere as left or right, or belonging to a sinistral or dextral person based only on its within-hemisphere connectivity. All three types of models almost perfectly identify the hemispheres in dextrals, with high success rates in sinistrals as well. To our knowledge, this is the first demonstration that hemisphere chirality can be identified from resting-state functional connectivity.

### Hemisphere identification

All the models performed well at classifying the hemispheres, but we investigated the LDAs further due to their interpretable parameters. In “hemisphere space,” we observe that, on average, sinistrals have hemispheres that are more similar to one another than dextrals do, although none are so similar that the LDA classifiers misclassify it. Secondly, generally speaking, using data collected during a language task (but treated as resting-state) did not improve performance, although this was likely attributable to the reduced acquisition time, and not a lack of task-induced connectivity changes. It is possible that connectivity changes induced in a longer task may prove helpful in distinguishing sinistrals and dextrals.

Our success in identification is supported by the literature identifying differences in structure (Sha et al., 2021) and structural connectivity between the hemispheres (Caeyenberghs & Leemans, 2014; D. Li et al., 2019; M. Li et al., 2014), including in non-human primates (Iturria-Medina et al., 2011). However, the differences in structure and task activation patterns (Szaflarski et al., 2002, 2012), do not seem to be reflected in functional connectivity to the degree required to independently categorize the hemispheres.

However, the difference between the hemispheres is not isolated to the language network, as one might predict from previous research on the topic. Rather, the strongest differences between the hemispheres are found in LN-DMN and -FPN connections. The FPN, DMN, and LN are highly related networks. Broadly speaking, the FPN regulates both the DMN and the LN (Gordon et al., 2020), and connectivity between these networks and between these networks and the rest of the brain is positively correlated with measures of intelligence (Marek & Dosenbach, 2018). Based on our findings, it bears investigation whether lateralization of these networks and their relationship to the LN are independently associated with intelligence. However, these networks are rarely discussed as lateralized (Cole et al., 2015; Hearne et al., 2016; Sheffield et al., 2015), although some authors have reported left-lateralization of the DMN and FPN (Agcaoglu et al., 2015).

The DMN, rather than the LN, was the sole network where the classifier relied on within-network connectivity to predict chirality, in dextrals, sinistrals and the dextral+sinistral analyses. The DMN and LN are strongly segregated from one another in task activations, an effect which is even stronger in RH compared to the LH (Mineroff et al., 2018). Although we do not investigate subnetworks of the large-scale RSNs in this analysis, the anterior subnetwork of the DMN preferentially connects to the LN, compared to the posterior (Gordon et al., 2020). This asymmetry in connectivity is reflected in broadly more efficient and less random organization in the LH compared to the RH (Caeyenberghs & Leemans, 2014; Dennis et al., 2013). These differences in efficiency are present from birth (Ratnarajah et al., 2013), which suggests genetic biases for hemispheric organization.

### Handedness identification

The distance between hemispheres scales with a continuous measure of handedness, with more sinistral participants (EHI < 70) having more similar hemispheres. This value suggests a neural basis for the cutoff between sinistral (or nondextral) and dextral brains. We did not find neural support for three handedness groups. In contrast, the psychometric literature has often found bases for dividing handedness into three groups (Dragovic, 2004; Papadatou-Pastou et al., 2020; Peters, 1992), but some authors find two groups (Büsch et al., 2010). For a two-group division (dextral vs. adextral), an EHI cutoff between +30 and +40 is common, see Przybylski and Kroliczak (2023) and citations within. Our value (+70) is much greater than this. But the lack of a second discontinuity between “sinistrals” and “mixed-handedness” is certainly affected by the rate of adextral participants in our sample.

That said, unlike hemispheres, which have no intermediate kinds, separating individuals with similar EHIs of, e.g., −5 and +5 into different groups may impair model performance. We did not test “strong handedness” groups, i.e. only sinistrals < −30 vs. dextrals > +30, in order to not decrease the size of the already-small (*n = 74*) sinistral training groups.

We did not find a U-shaped curve, where strong sinistrals pattern like strong dextrals. This lack of curve implies that greater separation between the hemispheres is associated with right-handedness, and not associated with a strong hand preference in general. That said, as with most resting-state studies (Marek et al., 2022), the effects are very small, explaining only a few percent of the variance.

Finally, from the hemisphere-by-handedness LDA, the second (Figure R7) and third linear discriminants provide hints that sinistrals are organized on one extreme of the norm, even if the pattern wasn’t sufficient to classify a connectome. It may be the case, therefore, that an analysis based on different methods of characterizing brain activity may be more fruitful in the classification problem. Using derived values, like graph-theoretic summary statistics (c.f. Caeyenberghs & Leemans, 2014) or differences between matched features may permit handedness classification.

### Analytic decisions

We used a single parcellation scheme that had some advantages, namely that there were the same (albeit non-homotopic) parcels in each hemisphere. Other schemes have found different parcels (in number and extent) in the two hemispheres. It may be possible that a different scheme might better capture variability, especially in RH, that is attributable to handedness. Likewise, using a single parcellation scheme for a large number of participants is a common analysis technique, but sinistrals are typified by their heterogeneity. As a result, a parcel applied to a sinistral brain may, on average, contain signal from more nearby, but unrelated regions than the same parcel applied to a dextral brain. As a consequence, the signal-to-noise ratio in a given parcel may be lower in sinistrals, harming model performance. As such, a de novo parcellation on each individual, with identity between parcellations in different individuals may permit classification.

### Future research

The creation of a scalar to measure the distance between hemispheres is advantageous for straightforward exploration of experiential effects on hemisphere organization. We propose three main avenues for future work in hemispheric distinctiveness. These areas are: the effects of motor training (i.e., handedness), hemispheric reorganization following stroke, and typical development.

Writing is especially interesting as a motor behavior because there is a multicultural history of teaching natural sinistrals to write with their non-dominant (i.e., right) hand in both the West (Guber, 2019; Kushner, 2012) and the East, especially when writing Chinese due to the stricter nature of logographic character stroke order (Kushner, 2013; Papadatou-Pastou et al., 2020). These “converted” sinistrals are aging, as the practice ended in the U.S. in roughly the 1970s (Hugdahl et al., 1993). However, there is structural (Klöppel et al., 2007, 2010) and motor-task evidence (Grabowska et al., 2012; Siebner et al., 2002) that this population differs cortically and subcortically from dextrals and non-converted sinistrals, primarily in the direction of increased bilaterality. Similarly, following dominant right-hand amputation, amputees show changes in control pathways and increase in LH activation for control of the non-amputated left hand (Philip & Frey, 2014). Applying this algorithm to these populations would also provide a natural experiment testing the effects of changes in motor dominance on cortex-wide organization, for example, are these individuals’ cortical organization much more like a strong dextral than their handedness would otherwise predict?

It also remains an open question how the uninjured hemisphere reorganizes after a stroke. DMN and FPN connectivity can be disrupted following a stroke, even if the lesion is outside of the network (Dacosta-Aguayo et al., 2015; Tuladhar et al., 2013), with changes in disruption patterns from the acute to chronic phase (Zhang, 2025). Conversely, damage to LH language areas nearly always causes aphasia, and damage to other regions, including the RH LN, does not. In people with aphasia, the RH becomes more involved in language processing, through increased recruitment of RH language regions, and recruitment of new RH areas, depending on lesion characteristics (Turkeltaub et al., 2025; Wilson & Schneck, 2021). Connections between the damaged LN and the FPN improve outcomes (Billot & Kiran, 2024). Our work shows that different patterns of LN-FPN and LN-DMN connections are among the most distinctive elements of the LH. As such, we can ask whether intact RHs tend to become more like LHs in the chronic period. While connectivity work has evaluated the connections between these networks, our protocol provides a simple, easily interpretable number (or set of numbers) that could be used to quantify “how much” an RH looks like a LH. Thus, we plan to apply this analysis to stroke connectomes to quantify gestalt hemispheric reorganization over recovery.

Finally, we also propose to use this model longitudinally. Is the “distance” between hemispheres innate at birth, i.e. differing by handedness groups, or does it emerge over time? Evidence from language laterality suggests that language is more bilateral in childhood (Olulade et al., 2020; Szaflarski, Holland, et al., 2006), thus we would expect the difference between the hemispheres to emerge over time, but the exact timing remains an empirical question. However, a developmental study could show the direction of the effect: Do more weakly lateralized brains at birth become left-handed, and strongly lateralized brains become right-handed? Or does a third factor determine whether weakly lateralized brains at birth (1) become distinct-and-dextral or (2) exhibit less change and become sinistral? This study would test whether there is a causal effect of handedness on brain organization.

While a decrease in bilaterality in development seems to occur, the reverse effect has been proposed in aging. This model is known as “hemispheric asymmetry reduction in older adults” (HAROLD; Cabeza et al., 2002), where functional lateralization decreases with time. While some studies have failed to support this hypothesis, e.g., Meunier et al. (2014), Nenert et al. (2017), our model could provide a simple test of this hypothesis in similar open datasets.

## Conclusions

We find that it is trivial to identify the chirality of a hemisphere given its functional connectivity patterns during rest. This classification relies on connections between the language network and attentional networks: default mode and frontoparietal networks, but not connections within the language network itself. In contrast, we could not reliably classify a hemisphere as belonging to a sinistral or dextral.

However, analyses of the difference *between* hemispheres suggests that there is an effect of handedness on hemispheric similarity, for all but the strongest right handers. With these findings, we can ask how the separation between the hemispheres emerges over development, does the distance increase with age, or is it a stable trait? This approach to quantifying the similarity between hemispheres we have described here opens up new avenues of research to study lateralization of the brain in development, aging, brain disorders, and recovery.

## Supplementary Material

Supplement 1

## Figures and Tables

**Figure R6:**
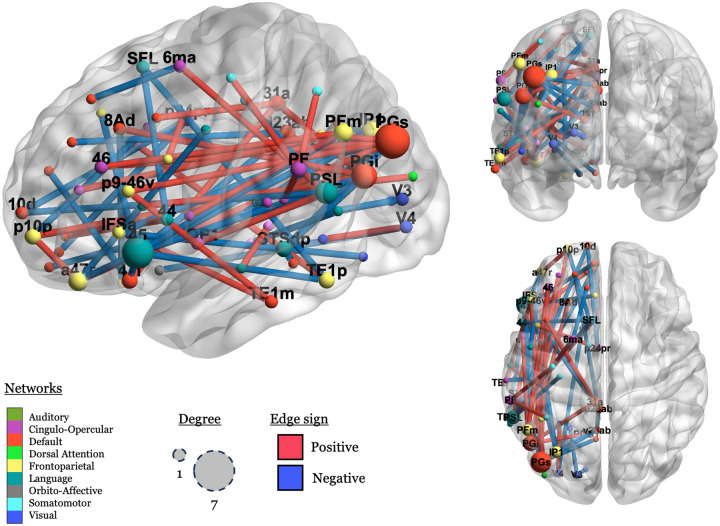
Visualization of significant connections and their associated nodes (ROIs) for the sinistral-only hemispheric classification analysis. Nodes (spheres) are scaled by the number of significant connections they are involved in (1 – 7), and nodes with degree > 1 are labeled. Edges are colored by sign (blue: negative; red: positive). Nodes are colored by network, using the colors used in Ji et al. (2019), with some modifications. Node names are from Glasser et al. (2016).

**Figure R6:**
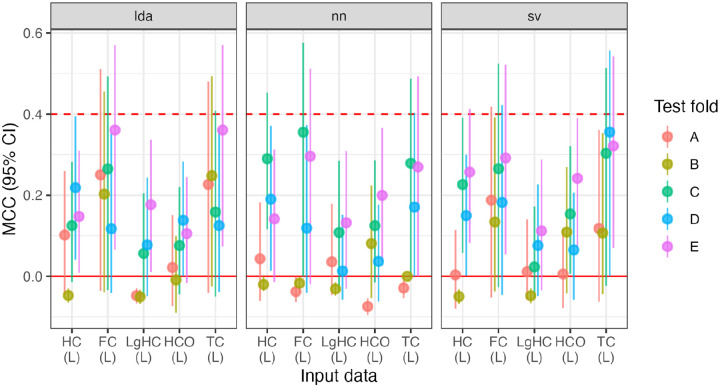
MCCs for the handedness prediction across folds and input data. HC: hemiconnectomes, FC: full connectome, LgHC: language connectome, HCO: oversampled hemiconnectomes, and TC: transconnectomes.

**Table M1:** Cole-Anticevic RSNs, by number of parcels in each hemisphere, and in sum. Abbreviations are given.

Network	Abbrv.	LH	RH	total
Auditory	AudN	8	7	15
Cingulo-opercular	CON	27	29	56
Default	DMN	40	37	77
Dorsal attention	DAN	12	11	23
Frontoparietal	FPN	22	28	50
Language	LN	14	9	23
* *Orbito-affective*	*OAN*	*3*	*3*	*6*
* *Posterior multimodal*	*PMN*	*3*	*4*	*7*
Somatomotor	SMN	19	20	39
* *Ventral multimodal*	*VMN*	*2*	*2*	*4*
Visual	VisN	3	3	6
Visual 2		27	27	54

*: small networks that are not analyzed further.

## Footnotes

1 Using the sklearn function SVC(), default settings other than using: a linear kernel and balanced class weights.

2 Using the sklearn function neural_network.MLPClassifier(), default settings, i.e., 100-layer.

3 Regions that are part of the LH LN, but a different network in the RH, by RH network: CON (region PSL), pMN (STV), FPN (44, IFSp), and DMN (STSda).

4
https://infer.tidymodels.org/reference/get_p_value.html
