# Supplementary material for: Identifying left and right hemispheres using functional connectivity
Source: bioRxiv. 2025 Dec 19:2025.12.12.694044. Originally published 2025 Dec 13. Preprint. [Version 2] doi: 10.64898/2025.12.12.694044 (PMC12710821; doi:10.64898/2025.12.12.694044)
Supplement: Supplement 1 [file media-1.pdf]

Supplementary Material YY: Parcel assignments

Thirteen parcels received different labels in the LH and RH in the Cole-Antecevic parcellation. They are detailed in ST YY.1

Supplementary Table YY.1: ROIs receiving different labels in each hemisphere.

**ROI**	**L Network**	**R Network**
PEF	Dorsal Attention	Cingulo Opercular
PSL	Language	Cingulo Opercular
STV	Language	Posterior Multimodal
33pr	Cingulo Opercular	Frontoparietal
d32	Default	Frontoparietal
44	Language	Frontoparietal
IFSp	Language	Frontoparietal
IFSa	Frontoparietal	Cingulo Opercular
OFC	Default	Frontoparietal
RI	Auditory	Somatomotor
STSda	Language	Default
31a	Default	Frontoparietal
TE1m	Default	Frontoparietal

Supplementary Material XX: Enrichment analyses

We repeat some sensitivity analyses for the main results of the right-handed analysis. First, SF XX.1 shows the results including the small excluded networks (OAN, v/pMN, see main text Table 2). The same pattern is found, and no significant connections or elevated significance rate are found in the excluded networks.

Following replacement of simulated *p* values of 0.0 with 0.003, and FDR correction for 66 multiple comparisons, the CON-DMN and DMN-FPN relationships are no longer significant (p_{cor} = *.19)*, but the other results remain significant at p_{cor} = *.0066.*

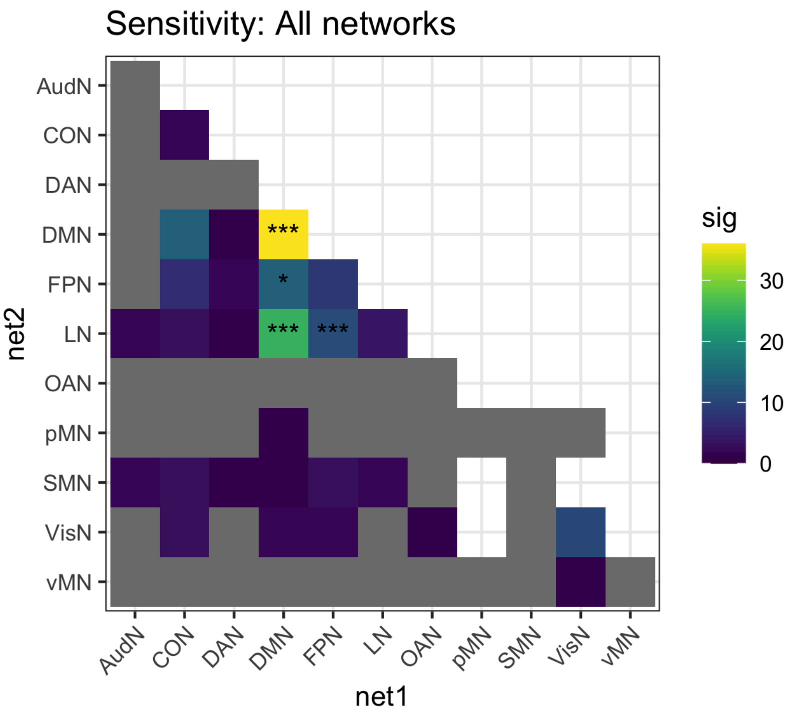

Supplementary Figure XX.1: Analyses repeated for the excluded networks (OAN, p/vMN).

Secondly, 13 parcels receive different labels in the LH and RH. As described in the main text, we used the LH labels where those labels conflicted. SF XX.2 shows the same analysis, but with those 13 parcels removed. Similar patterns are found, with the exception that CON-DMN connections are also implicated when those parcels are removed. Nine out of those 13 parcels are assigned to either CON or DMN in either hemisphere, see ST YY.1.

Following replacement of simulated p values of 0.0 with 0.003, and FDR correction for 36 multiple comparisons, the CON-DMN and DMN-FPN relationships are no longer significant ($p_{cor} = .15$), but the other results remain significant at $p_{cor} = .0036$.

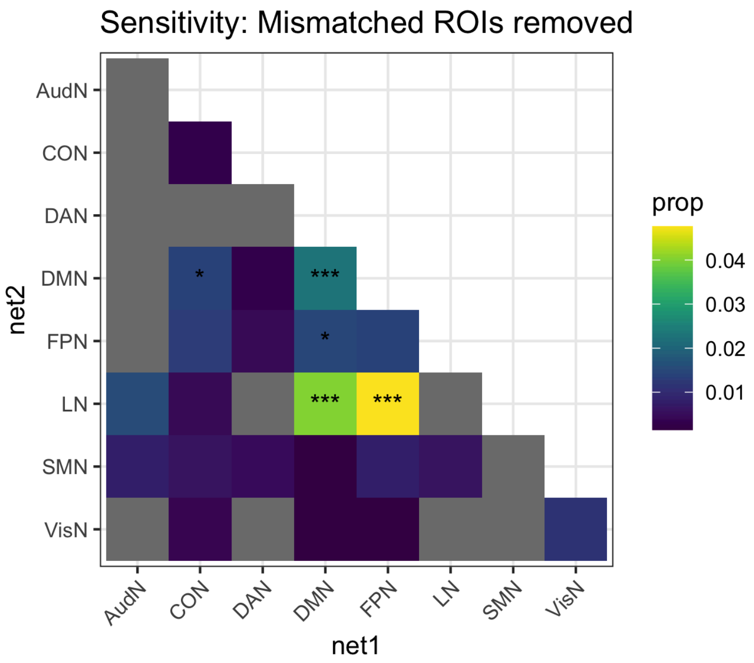

Supplementary Figure XX.2: Analyses repeated with the 13 parcels with mismatched left and right networks removed.

We repeat the regular analysis for sinistrals only, i.e., maintaining network exclusions, and not excluding mismatched parcels. Compare this plot to the main text Figure R3(C). After *p*-value replacement and FDR correction for multiple comparisons, the within-FPN effect is not significant ($p = .18$), but the rest remain significant at $p = .0036$.

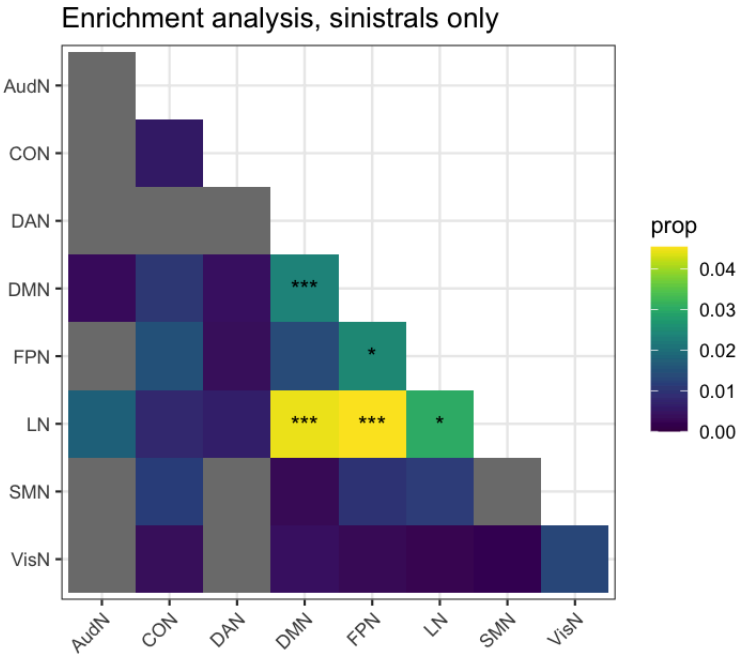

Supplementary Figure XX.3: Which network pairs (or single networks) have more significant connections than expected by chance. Following main text Figure R3(C).

We repeat the regular analysis for the four-way analysis, i.e., maintaining network exclusions, and not excluding mismatched parcels. Following *p*-value replacement, and FDR correction for 36 multiple comparisons (within each LD), the CON-DMN result is no longer significant for LD3, $p_{cor} = .24$, but the DMN-LN connection remains significant, $p_{cor} = .01$. All LD1 results remain significant at $p = .0036$..

The results of this analysis are very similar to the single-handedness-group results, except for the addition of LN-AudN connections in classifying the hemispheres that was not present previously.

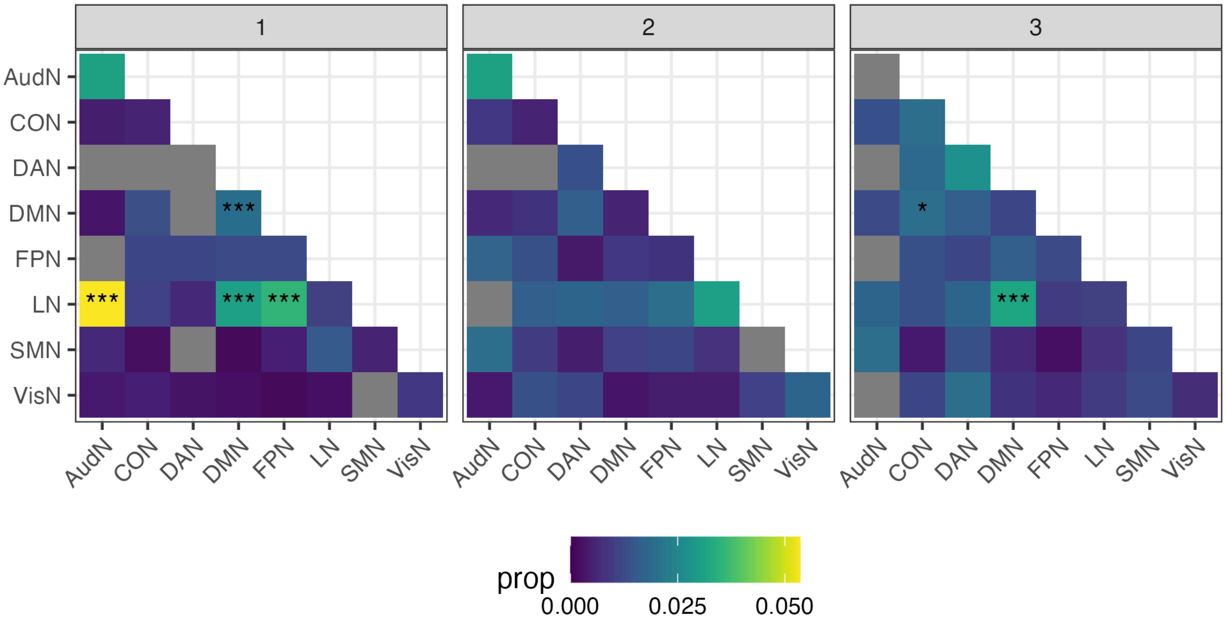

Supplementary Figure XX.4: The same analyses repeated for the three LDs resulting from the four-way classification.

In order to interpret Figure R5, we plot the LD1 connection weights against the underlying difference in connectivity between the hemispheres (LH-RH). For significant connections, greater magnitudes are associated with less difference between the hemispheres, whereas the smallest magnitudes are associated with greater differences.

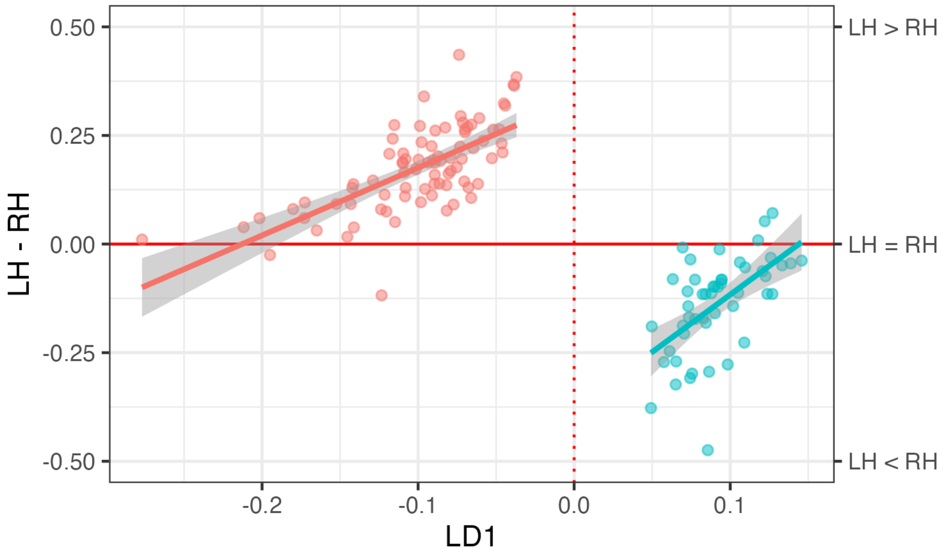

Supplementary Figure XX.5: The relationship between LD1 values for the dextral analysis (*x* axis) and the absolute difference in connections (*r* values), *LH - RH*.

Supplementary Material ZZ: MCC

In order to demonstrate the differences between accuracy and MCC, take the following example. There are 200 dextral (91%) and 20 sinistral (9%) individuals in our sample. As we iterate from 0/20 correctly predicted sinistrals to 20/20 correctly predicted sinistrals, the confusion matrix (Table SM1.1) changes. For this example, the number of correctly predicted dextrals is held constant.

Table SM ZZ.1: Example confusion matrices, for (left) 100% assignment for dextrals and changing sinistral success ratio and (right) vice versa for changing dextral success ratio.

Changing sinistral ratio					Changing dextral ratio			
		Predicted class					Predicted class	
		Sinistral	Dextral				Sinistral	Dextral
True class	Sinistral	*x*	*20-x*		True class	Sinistral	20	0
	Dextral	0	200			Dextral	*200-x*	*x*

For "changing sinistral ratio," as x increases from 0 to 20, accuracy increases from $\frac{200}{220} = 0.91$ to 1.00, but MCC increases from 0 to 1. But, for example, a 50% error rate in the sinistral group leads to an MCC of 0.69, but an accuracy of 0.95.

For "changing dextral ratio," as x increases from 0, 200, accuracy increases from $\frac{20}{220} = 0.09$ to 1.00, and MCC increases from 0 to 1. However, as can be seen in Figure SM1.1, MCC is not completely insensitive to class size: A 50% error rate in the larger dextral group leads to an MCC of 0.29 (compared to *MCC = 0.69* above), compared to an accuracy of 0.54.

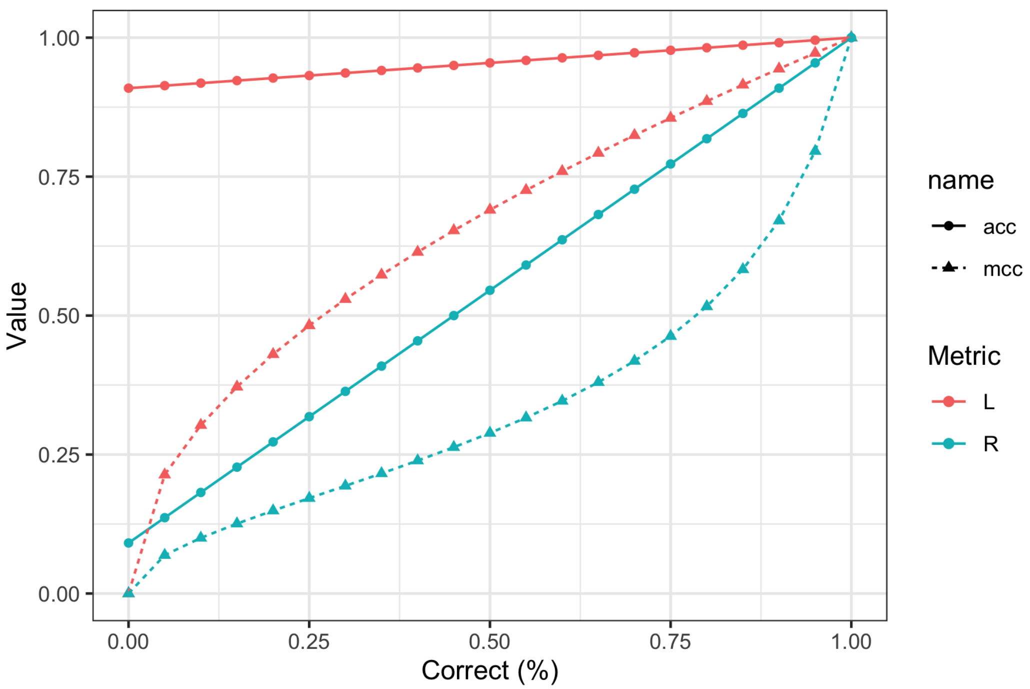

Figure SM ZZ.1: Changes in accuracy (solid lines) and MCC (dashed lines) as the number of correctly predicted sinistrals (red) or dextrals (blue) changes, with the other group held constant at 100%.

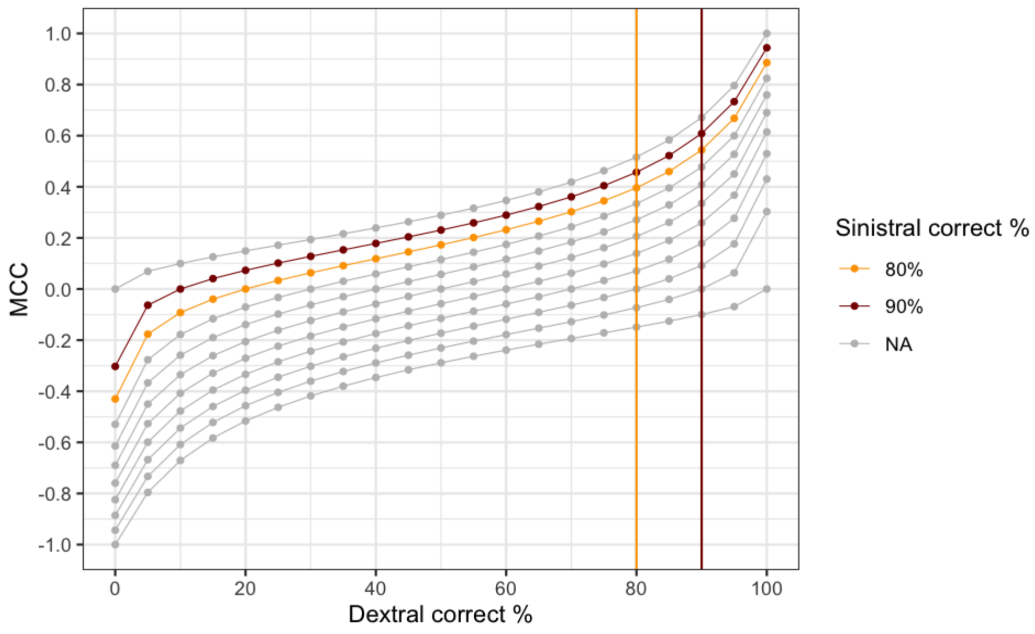

Figure SM ZZ.2: Simulated MCC values (y-axis) for various values of dextral correct assignment rates (x-axis), by various values of sinistral correct assignment rates (lines). A correct assignment rate of 80% in each group is colored orange, and 90%, red.

Supplementary Material AA: Quadratic modeling

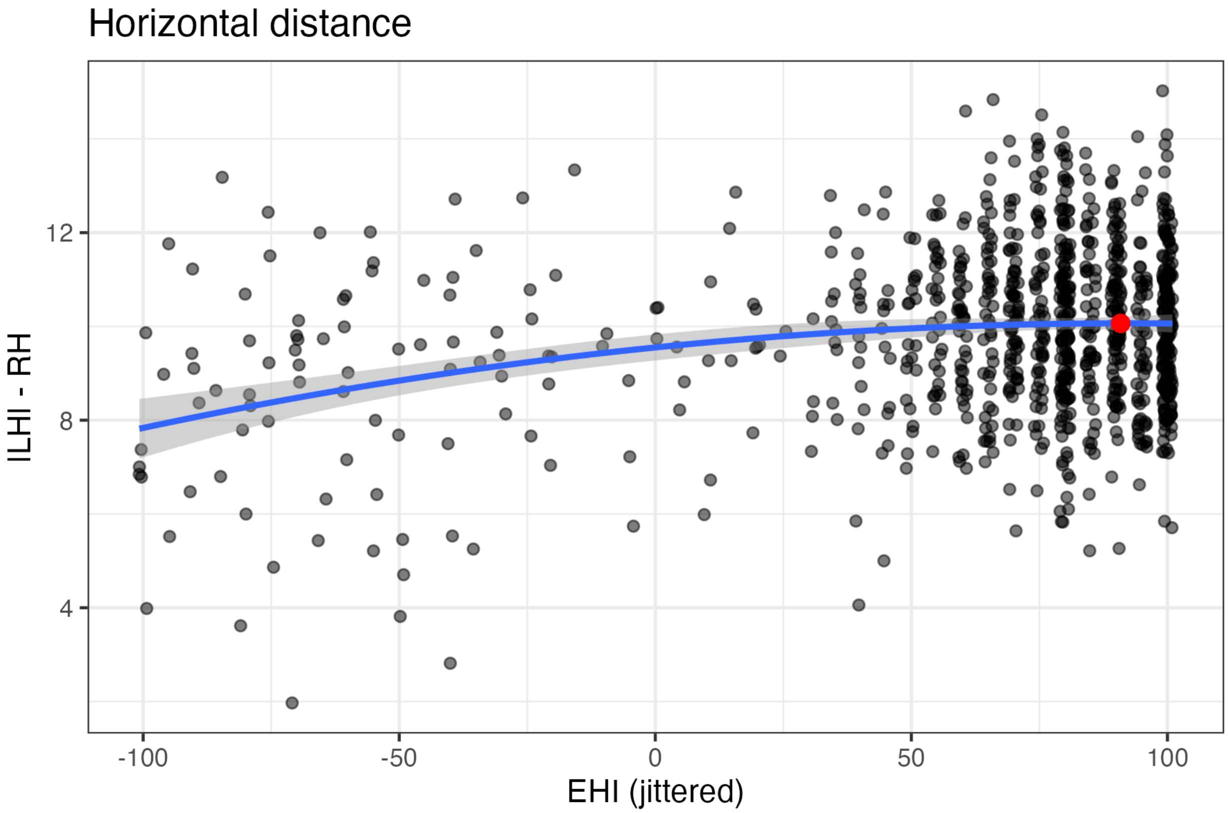

Supplementary Figure AA.1: The quadratic model for the relationship between hemisphere distance and EHI.

The quadratic model discussed in the main text as an alternative to the segmented linear regression. Where the segmented linear regression finds a breakpoint at EHI = 75, this model finds a peak at EHI = 91.
